# Electrocardiographic features of disease progression in arrhythmogenic right ventricular cardiomyopathy/dysplasia

**DOI:** 10.1186/1471-2261-15-4

**Published:** 2015-01-19

**Authors:** Ardan M Saguner, Sabrina Ganahl, Andrea Kraus, Samuel H Baldinger, Deniz Akdis, Arhan R Saguner, Thomas Wolber, Laurent M Haegeli, Jan Steffel, Nazmi Krasniqi, Thomas F Lüscher, Felix C Tanner, Corinna Brunckhorst, Firat Duru

**Affiliations:** Department of Cardiology, University Heart Center Zurich, Rämistrasse 100, CH-8091 Zurich, Switzerland; Division of Biostatistics, Institute for Social and Preventive Medicine, University Zurich, Zurich, Switzerland; Department of Cardiology, University Hospital Bern, Bern, Switzerland; Center for Integrative Human Physiology, University Zurich, Zurich, Switzerland

**Keywords:** Arrhythmogenic Right Ventricular, Cardiomyopathy, Dysplasia, Electrocardiography, T wave inversion

## Abstract

**Background:**

Arrhythmogenic right ventricular cardiomyopathy/dysplasia (ARVC/D) is considered a progressive cardiomyopathy. However, data on the clinical features of disease progression are limited. The aim of this study was to assess 12-lead surface electrocardiographic (ECG) changes during long-term follow-up, and to compare these findings with echocardiographic data in our large cohort of patients with ARVC/D.

**Methods:**

Baseline and follow-up ECGs of 111 patients from three tertiary care centers in Switzerland were systematically analyzed with digital calipers by two blinded observers, and correlated with findings from transthoracic echocardiography.

**Results:**

The median follow-up was 4 years (IQR 1.9–9.2 years). ECG progression was significant for epsilon waves (baseline 14% vs. follow-up 31%, p = 0.01) and QRS duration (111 ms vs. 114 ms, p = 0.04). Six patients with repolarization abnormalities according to the 2010 Task Force Criteria at baseline did not display these criteria at follow-up, whereas in all patients with epsilon waves at baseline these depolarization abnormalities also remained at follow-up. T wave inversions in inferior leads were common (36% of patients at baseline), and were significantly associated with major repolarization abnormalities (p = 0.02), extensive echocardiographic right ventricular involvement (p = 0.04), T wave inversions in lateral precordial leads (p = 0.05), and definite ARVC/D (p = 0.05).

**Conclusions:**

Our data supports the concept that ARVC/D is generally progressive, which can be detected by 12-lead surface ECG. Repolarization abnormalities may disappear during the course of the disease. Furthermore, the presence of T wave inversions in inferior leads is common in ARVC/D.

## Background

Arrhythmogenic right ventricular cardiomyopathy/dysplasia (ARVC/D) is an inherited heart muscle disorder characterized by fibro-fatty infiltration of the right ventricle (RV) [1, 2]. The disease is a common cause of sudden cardiac death among young adults and athletes [3–5]. ARVC/D can manifest with multiple facets ranging from primarily ventricular tachyarrhythmia to biventricular heart failure that may eventually lead to heart transplantation or cardiac death [6, 7].

The diagnosis of ARVC/D according to the 2010 Task Force Criteria (TFC) is based on six categories with major and minor criteria in each. These categories comprise: 1. Global or regional dysfunction and structural alteration of the RV visualized by echocardiography, cardiac magnetic resonance tomography, or RV angiography 2.Tissue characterization of the RV free wall by endomyocardial biopsy 3. 12-lead surface electrocardiographic (ECG) repolarization abnormalities 4. ECG depolarization abnormalities 5. Ventricular tachyarrhythmias 6. Family history/genetic testing. The diagnosis is possible if 2 minor criteria or 1 major criterion are present. It is borderline if 1 major and 1 minor or 3 minor criteria are present, and it is definite if 2 major or 1 major plus 2 minor or 4 minor criteria are present [6, 7].

Recent data suggests that diagnostic features of ARVC/D such as epsilon waves and T wave inversions (TWI) may disappear on serial ECG evaluations during follow-up [8], although ARVC/D is generally considered an irreversible and progressive cardiomyopathy [9–11]. TWI in inferior leads have recently been proposed as valuable diagnostic and prognostic markers in this entity, but have not been incorporated in the TFC [12, 13]. Whereas TWI in V1- V2 or in V4, V5, or V6 are a minor criterion for ARVC/D, epsilon waves constitute a major criterion owing to the fact that epsilon waves are rather specific for ARVC/D. Precordial TWI are more sensitive, but lack specificity as they are often present in other diseases such as ischemic or congenital heart disease. Moreover, precordial TWI in V1-V3 and inferior lead TWI can be observed in a minority of healthy probands [14]. Since data on clinical features of disease progression are limited, and correlations between inferior lead TWI and other diagnostic parameters have not been well defined in ARVC/D, the aim of this study was to identify associations between inferior lead TWI, other ECG variables, and echocardiographic findings, and to assess ECG changes during long-term follow-up in a large cohort of patients with ARVC/D [14–16].

## Methods

### Study population

The study population included 111 patients from three tertiary-care centers in Switzerland with a definite (n = 80), borderline (n = 19), and possible (n = 12, all first degree family members of patients with confirmed ARVC/D) diagnosis of ARVC/D according to the 2010 TFC, who had an ECG recorded between February 1987 and March 2013 [7]. This baseline ECG was the first available ECG. Clinical information regarding demographics and symptoms were obtained from hospital records at the time of the baseline ECG. Coronary ischemia was ruled out in the presence of precordial TWI. If patients with precordial TWI presented with typical angina, or were >35 years old and presented with ventricular tachycardia or arrhythmogenic syncope, coronary angiography was performed to rule out significant coronary artery disease. In all other patients non-invasive tests such as cardiac MRI, myocardial perfusion scintigraphy, stress TTE or treadmill stress testing were performed to rule out coronary ischemia. This study was approved by the Ethics Committee of the Canton of Zurich and the Ethics Committee of the Canton of Bern, Switzerland, and has been performed in accordance with the ethical standards laid down in the 1964 Declaration of Helsinki and its later amendments. Patients gave written informed consent for prospective inclusion in this study.

### Definitions of 12-lead surface ECG and transthoracic echocardiography parameters

ECG and transthoracic echocardiography (TTE) definitions and criteria used in this study are presented in Table 1.

**Table 1:** **Definitions of electrocardiographic and echocardiographic parameters used in this study**

Variables	Definitions
Epsilon wave (major depolarization criterion)	Distinct waves of small amplitude that occupy the ST segment in right precordial leads (V1-V3) and are distinct from the QRS complex
Terminal activation duration (minor depolarization criterion)	Longest value in V1 through V3 from the nadir of the S wave to the end of all depolarization
T wave inversion	Any T wave negativity
Inferior leads T wave inversion	Inverted T waves in 2 out of 3 inferior leads (II, III, and aVF)
Major repolarization criteria	Inverted T waves in right precordial leads (V1, V2, and V3) or beyond in individuals >14 years of age (in the absence of complete right bundle branch block)
Minor repolarization criteria	- Inverted T waves in leads V1 and V2 in individuals >14 years of age (in the absence of complete right bundle branch block) or in V4, V5, or V6
	- Inverted T waves in leads V1, V2, V3, and V4 in individuals >14 years of age in the presence of complete right bundle branch block
Complete right bundle branch block	QRSd ≥120 ms and:
	A1: R’ or r’ in V1 or V2
	A2: S duration > R duration in I and V6
	A3: S duration >40 ms in I and V6
	A4: R peak time >50 ms in V1 or V2
	a: A1 + A2
	b: A1+ A3
	c: A4+ (A2 or A3)
Incomplete right bundle branch block	QRS <120 ms and R peak time in V1 or V2 > 50 ms
QRS notching	Additional deflections/notches at the beginning of the QRS, on top of the R-wave, or in the nadir of the S-wave in the absence of bundle branch block
Left ventricular involvement	Echocardiographic documentation of LV wall motion abnormalities or a reduced ejection fraction (<50%) in the absence of other causes
Right ventricular regional wall motion abnormalities	Echocardiographic documentation of regional right ventricular akinesia/dyskinesia

### ECG analysis

ECGs were recorded at rest (25 mm/s, 10 mm/mV amplitude) with standard lead positions, digitized with a high-resolution scanner, and analyzed with a digital caliper (Iconico; screen caliper version 4.0; http://www.iconico.com) [15]. To increase the accuracy of measurements, all ECGs were enlarged four times. ECG intervals were measured in two consecutive sinus beats in each lead; the mean value of the two beats was used. If the difference between the two beats was >10 ms, then the mean of three beats was taken. Each ECG was independently analyzed by two blinded experienced readers. Differences in the interpretation of the ECG parameters were adjudicated by a third cardiologist, and a final conclusion was made by consensus. ECG variables were measured as previously described [13].

### Transthoracic Echocardiography

Conventional M-mode, 2-D, and color Doppler echocardiography was performed in 111 patients at the time of the baseline ECG by experienced cardiologists according to guidelines [16, 17]. Left ventricular (LV) involvement was diagnosed when regional wall motion abnormalities or a reduced ejection fraction (EF <50% by Simpson’s biplane method) were present, and other causes were excluded [18]. Some ECG data from 90 patients and TTE data from 70 patients used in this study has been previously reported by our group [11, 13]. While we focused on predictors of adverse outcome in these previous studies, the current study investigates ECG changes during long-term follow-up, and their associations with TTE findings. Thus, data on follow-up ECGs have not been previously reported by our group.

### Statistical analysis

Continuous variables are presented as mean ± SD or median (with interquartile ranges, IQR), and were compared using a two-sided t-test or Mann–Whitney U-test, as appropriate. Categorical variables are reported as frequency (percentage) and compared between groups by Fisher’s exact test or χ^2^ –test of independence, as appropriate. Logistic univariate regression analysis was used to correlate between TWI in inferior leads as the response, and number of precordial leads with TWI as the explanatory variable. For comparison of ECG data between baseline and follow-up, we used the Wilcoxon signed-rank test for continuous variables, and Mc Nemar’s test for categorical variables. A two-sided p-value of ≤0.05 was considered significant. Statistical analysis was performed using R programming language (R Development Core Team, 2009) and GraphPad Prism 5 (GraphPad Software Inc., La Jolla, CA, USA).

## Results

### Baseline characteristics

Patient baseline characteristics are summarized in Table 2. A baseline ECG was available in 111 patients. TTE at the time of the baseline ECG was performed in all 111 patients. Ninety-eight patients were index patients, and 13 were family members. Sixty-four (58%) patients had an implantable cardioverter-defibrillator. ECG did not display abnormal findings in 14 patients (13%). Epsilon-waves were most frequently observed in V1 (14%). Epsilon-like waves in any lateral precordial lead (V4, V5, or V6) were present in a minority of patients (5%), with one patient displaying these potentials in V2 through V6. Complete right bundle branch block (RBBB) was observed in 14 (13%), incomplete RBBB in 3 (3%), complete left bundle branch block in 2 (2%), left anterior fascicular block in 7 (6%), and unspecific QRS notching in 29 (26%) patients. On echocardiography, regional wall motion abnormalities were most commonly observed in the RV subtricuspid region (n = 66; 60%).

**Table 2:** **Baseline characteristics**

Patient characteristic	All patients (n = 111)
Male, n (%)	71 (64%)
Age at baseline ECG (years)	43 (30–56)
Systolic blood pressure (mmHg)	120 ± 19
Diastolic blood pressure (mmHg)	76 ± 9
Heart rate (bpm)	65 (57–74)
Body mass index (kg/m^2^)	24.3 ± 3.2
Medication	
Amiodarone, n (%)	17 (15%)
Beta-blocker, n (%)	47 (42%)
Sotalol, n (%)	13 (12%)
12-lead surface ECG	
Epsilon waves in V1, V2, or V3	21 (19%)
Minor ECG depolarization criteria (2010 TFC)	25 (23%)
Terminal activation duration (ms)	52 (43–64)
Major ECG repolarization criteria (2010 TFC)	37 (33%)
Minor ECG repolarization criteria (2010 TFC)	21 (19%)
TWI in inferior leads	38 (34%)
TWI in V4, V5, or V6	43 (39%)
Number of leads with TWI	4.5 ± 2.5
Number of precordial leads with TWI	2.6 ± 1.9
QRS duration (ms)	111 (100–116)
Corrected QT duration (ms)	444 (426–480)
Transthoracic echocardiography	
Patients with RV regional wall motion abnormalities	85 (77%)
1 RV region involved	25 (23%)
≥2 RV regions involved	56 (50%)
Patients with LV involvement	15 (14%)

*Abbreviations*: *ARVC/D* Arrhythmogenic right ventricular cardiomyopathy/dysplasia, *LV* left ventricular, *RV* right ventricular, *TFC* 2010 Revised ARVC/D Task Force Criteria, *TWI* T wave inversions.

Values are means ± standard deviation, medians with interquartile ranges and numbers (percentages).

### 12-lead ECG changes during follow-up

**Figure 1:**
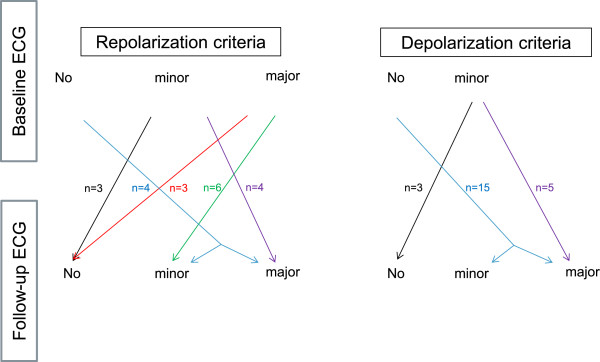
**Illustrative diagram of changes in depolarization and repolarization criteria during follow-up in patients with ARVC/D.** Patients not presented in this figure remained in the same category of depolarization and repolarization criteria at baseline and follow-up.

**Figure 2:**
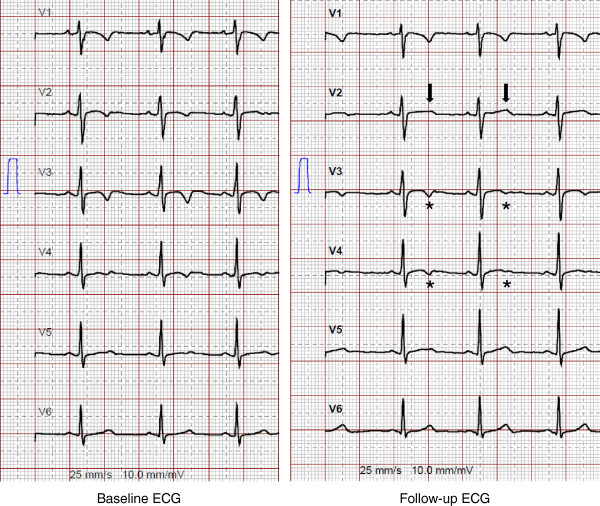
**Representative 12-lead surface ECG (25 mm/s, 10 mm/mV) from a patient with ARVC/D displaying major repolarization 2010 task force criteria at baseline (left panel), and minor repolarization criteria (in the absence of complete RBBB) at follow-up due to normalization of T waves in lead V2 (black arrows, right panel).** This variation may have been caused by slightly different fixation of V2, although R/S transition occurs in V3 in both ECGs. Also note the beat-to-beat variation of T wave morphology in leads V3 and V4 (asterisks).

**Figure 3:**
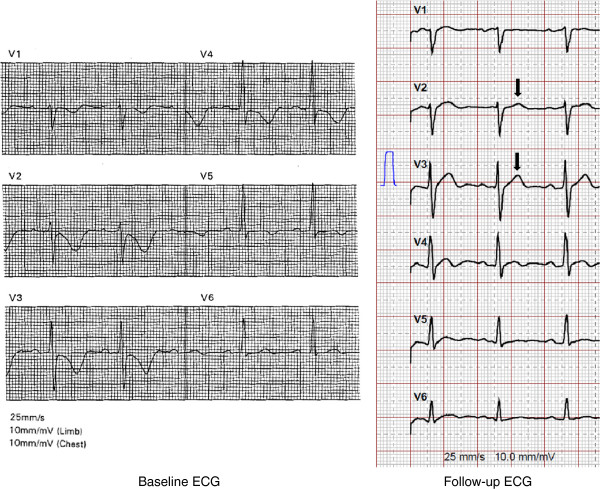
**Representative 12-lead surface ECG (25 mm/s, 10 mm/mV) from a patient with ARVC/D and dynamic repolarization abnormalities, presumably T wave memory.** The left panel shows major repolarization abnormalities according to the 2010 task force criteria at baseline, but no repolarization criteria at follow-up with normalization of T waves in leads V1-V5 (right panel, black arrows). This patient suffered from sustained ventricular tachycardia from the RV outflow tract, and imaging revealed akinesia of the RVOT corresponding to scar visualized by electroanatomical voltage mapping.

A follow-up ECG was available in 77 patients. In the remaining 34 patients, a follow-up ECG was not obtained due to short follow-up periods (n = 14) or loss of follow-up/heart transplantation/death (n = 20). The median time between baseline and follow-up ECG was 4 years (IQR 1.9-9.2 , range 0.3–21.8 , mean 5.6 years). The follow-up ECG was considered normal in 9 patients (12%). On average, ECG findings were progressive during follow-up, although individual disease course showed some variations. This progression was statistically significant for major depolarization criteria, which was particularly attributed to an increase in patients with epsilon waves in V2 at follow-up, and an increase in the maximum QRS duration (Table 3). More patients had TWI in the precordial leads at follow-up. Out of 8 patients without any precordial TWI at baseline, 4 (50%) later displayed precordial TWI. An illustrative diagram of electrocardiographic de- and repolarization abnormalities at baseline and their changes during follow-up is shown in Figure 1. Three patients with minor repolarization criteria at baseline did not have any repolarization criteria at follow-up. Four patients with major repolarization criteria at baseline had minor repolarization criteria in the absence of complete RBBB at follow-up (Figure 2), whereas two patients with major repolarization criteria displayed minor repolarization criteria in the presence of complete RBBB at follow-up. Repolarization criteria were completely absent in three patients, who had major repolarization criteria at baseline (Figure 3).

**Table 3:** **ECG variables in 77 patients, in whom a baseline and follow-up 12-lead surface ECG was available**

ECG variable	Baseline ECG	Follow-up ECG	p-value
Epsilon waves in V1, V2, or V3	11 (14%)	24 (31%)	0.01
Late potentials in inferior leads	6 (8%)	9 (12%)	0.45
Minor ECG depolarization criterion	21 (27%)	19 (25%)	0.56
Terminal activation duration (ms)	50 (44–64)	50 (45–60)	0.47
Major ECG repolarization criterion	29 (38%)	28 (36%)	0.58
Minor ECG repolarization criterion	14 (18%)	18 (23%)	0.55
T wave inversions in inferior leads	28 (36%)	31 (40%)	0.65
T wave inversions in V4, V5, or V6	33 (43%)	40 (52%)	0.25
Number of leads with T wave inversions	4 (2-6)	5 (2–7)	0.18
Number of precordial leads with T wave inversions	2 (1–4)	3 (1–5)	0.1
QRS duration (ms)	111 (100–125)	114 (100–128)	0.04
QT duration (ms)	431 (399–435)	420 (400–441)	0.57

Values are means ± standard deviation, medians with interquartile ranges and numbers (percentages).

**Figure 4:**
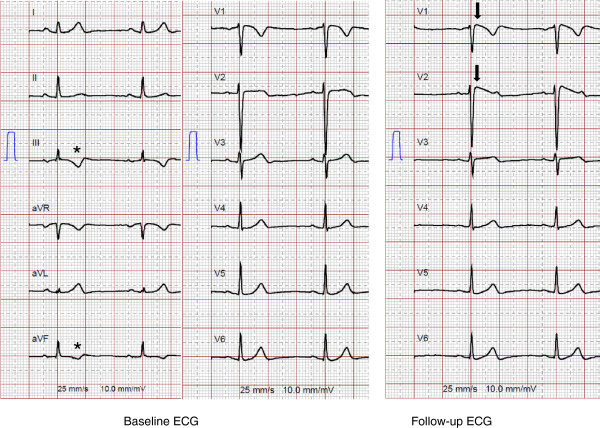
**12-lead surface ECG (25 mm/s, 10 mm/mV) from a patient with ARVC/D and a dynamic Brugada-like ECG pattern (criteria for Brugada Type I ECG not fulfilled).** The right panel shows a coved-type Brugada-like ECG in leads V1 and V2 at follow-up (black arrows), which is not visible at baseline (left panel). Also note the T wave inversions in the inferior leads (III and aVF) in the absence of repolarization criteria (asterisks).

From the patients without epsilon waves at baseline, 19% had these abnormalities at follow-up. In all patients displaying epsilon waves at baseline these potentials also remained at follow-up. In one patient, follow-up ECG revealed a dynamic Brugada-like ECG pattern (Figure 4). Epsilon-like waves in V4, V5 or V6 were present in a higher proportion of patients at follow-up compared to baseline (9%). Median terminal activation duration (TAD) at follow-up was similar compared to baseline. An increase in the number of precordial leads with TWI was particularly attributed to new TWI in leads V4 and V5 at follow-up compared to baseline (49% vs. 36% for V4, and 38% vs. 26% for V5, respectively). At follow-up, complete RBBB was observed in 12 (16%), incomplete RBBB in 2 (3%), complete left bundle branch block in 2 (3%), left anterior fascicular block in 5 (6%), and unspecific QRS notching in 23 (30%) patients. The maximum corrected QT interval did not significantly differ between baseline and follow-up.

### Transthoracic echocardiographic findings at follow-up

TTE data was available in all 77 patients at the time of the follow-up ECG. Echocardiographic data also indicated disease progression, and correlated with the ECG findings. Accordingly, LV involvement was present in 20 patients at follow-up (26% vs. 14% at baseline, p = 0.04). At follow-up, 15 (19%) patients remained with only one RV region involved, whereas 48 patients (62%) had ≥2 RV regions involved (p = 0.14 compared to baseline).

### Association between ECG and TTE findings

TWI in inferior leads were significantly associated with major repolarization criteria (p = 0.02), extensive RV involvement (p = 0.04), the presence of TWI in either lateral precordial lead (V4, V5 or V6; p = 0.05), and the presence of definite ARVC/D (p = 0.05), whereas the presence of echocardiographic LV involvement (p = 0.11) did not reach statistical significance. Regional wall motion abnormalities of the subtricuspid region were not significantly associated with TWI in inferior leads (p = 0.16). Univariate logistic regression revealed a highly significant incremental association between the absolute number of precordial leads with TWI and TWI in inferior leads (odds ratio 1.45, 95% confidence interval 1.16 - 1.83, p = 0.001).

## Discussion

This observational study constitutes the largest series investigating ECG changes during long-term follow-up in patients with ARVC/D, and has three main findings: First, our study supports the concept that ARVC/D is generally progressive in nature, and that surface 12-lead ECG is able to detect disease progression. Second, precordial T wave inversions may disappear during the disease course, whereas epsilon waves persist; and third, the presence of TWI in inferior leads is common.

### Previous studies - disease progression

Except for one study [19], four previous studies have suggested that ECG changes are generally progressive in patients with ARVC/D. Yet, the extent and speed of disease progression shows some individual variability that may be based on genetic and environmental factors [9–11, 20–22].

### Previous studies – dynamic ECG changes

Recent data suggests that electrocardiographic diagnostic features of ARVC/D such as epsilon waves and TWI may be dynamic and transient [8], which may potentially impede appropriate diagnosis of ARVC/D at certain time points during the disease course, particularly at early stages.

### Our findings

This study focused on patients with less advanced disease in comparison to previous studies analyzing data from patients with more advanced forms of ARVC/D, and underscores that disease progression can be visualized by consecutive ECG tracings, although the disease course shows some individual variations. Importantly, the percentage of patients with epsilon waves and average QRS duration significantly increased over time, and the increase in the number of precordial leads with TWI showed a tendency towards significance. With regard to TWI, the percentage of patients with precordial TWI beyond V3 and TWI in inferior leads increased, and correlated well with more extensive echocardiographic RV involvement and new onset LV involvement at follow-up. ARVC/D has been recently recognized as a biventricular disease with early LV involvement [23]. With respect to this, repolarization abnormalities beyond V1-V3, but also inferior lead TWI may serve as early ECG markers of biventricular involvement and widespread disease. The fact that depolarization abnormalities appear to better reflect disease progression compared to TWI is not completely clear. It is possible that repolarization abnormalities are not only caused by structural myocardial changes, but may also constitute a physiological variant [14], may vary depending on exercise status of a given patient [24], be caused by prolonged bouts of paroxysmal tachyarrhythmia or frequent ventricular premature contractions, i.e. T wave memory [25], and subtle differences in positioning the precordial electrodes, i.e. ECG reproducibility [26]. Anatomic predisposition, particularly in women, may affect lead positioning, and thus alter the presence and distribution of leads with TWI (Figure 3). Of note, in this study all four patients with major repolarization criteria at baseline, who presented with minor repolarization criteria in the absence of complete RBBB at follow-up, were women [8]. We were also able to document a dynamic Brugada-like ECG pattern in leads V1/V2 in one patient, as previously reported, although the criteria for Brugada Type I ECG were not fulfilled in the absence of J-point elevation ≥2 mm and TWI in V2 [27, 28]. Interestingly, this patient was one out of 13, in whom TWI in inferior leads were documented in the absence of major and minor repolarization criteria (34% of patients with TWI in inferior leads). In contrast to the study by Quarta et al. [8], dynamic ECG changes in our study primarily originated from changes in repolarization rather than depolarization abnormalities. Furthermore, as opposed to that previous study, all three patients with major repolarization criteria at baseline and absence of any repolarization criteria at follow-up had advanced forms of ARVC/D, suggesting that dynamic repolarization abnormalities may not only affect early forms of the disease.

Based on our data, depolarization abnormalities seem to be far less prone to reversal, because they probably better reflect myocardial infiltration by fibro-fatty tissue. This interpretation is further supported by our data on QRS duration, which also significantly increased during follow-up. As the proportion of patients with antiarrhythmic medication were not significantly different at baseline and follow-up, and TAD did not change over time, this increase can be attributed to a progression in depolarization abnormalities also affecting early depolarization such as bundle branch block and QRS notching. As such, the percentage of patients with RBBB and QRS notching increased during follow-up.

### TWI in inferior leads

The presence of TWI in lateral leads in patients with ARVC/D is well established, and a noninvasive indicator of biventricular involvement and severe RV dilation [29–32]. However, data about TWI in inferior leads in ARVC/D is scarce and their role not completely understood. Whereas most studies support the concept that TWI in inferior leads are a marker of progressive disease with extensive RV involvement and LV involvement [31, 32], other studies suggest that such ECG changes could also be associated with isolated wall motion abnormalities of the subtricuspid region. Steriotis et al. have recently reported a considerable prevalence of TWI in inferior leads in ARVC/D, particularly in the presence of severe RV dilation, but did not report on the prevalence of LV involvement in their cohort. Our study confirms these previous findings with more than one third of patients displaying TWI in inferior leads at baseline – far more than depolarization abnormalities in these leads. We further add to the concept that inferior lead TWI in ARVC/D are associated with extensive disease as reflected by a significant association with definite disease status, extensive RV involvement, and TWI in lateral precordial leads, and a tendency towards significance for LV involvement. On the contrary, we did not observe a significant association between inferior lead TWI and isolated regional wall motion abnormalities of the subtricuspid region. TWI in inferior leads could even constitute markers for widespread biventricular disease, especially in the presence of major repolarization abnormalities implying that TWI in inferior leads may have a rather non-benign character. Based on our findings, we believe that ECGs obtained once yearly can serve as an informative, cheap and readily available monitoring tool. In case of progressive ECG findings, further more expensive diagnostic tests such as echocardiography, cardiac MRI or Holter monitoring can be performed, and follow-up strategies can be refined. In the absence of ECG changes and a clinical deterioration during follow-up, in our routine clinical practice we perform TTE every 2 years, and cardiac MRI every 2–5 years to monitor disease progression. Yet, as an isolated diagnostic test ECG may not drive therapy per se, and ECG findings have to be incorporated in the whole clinical picture, especially when arrhythmic episodes are lacking. Accordingly, as a sole diagnostic method, ECG is not sensitive and specific enough to establish the diagnosis of ARVC/D. Therefore, other diagnostic tests including imaging (e.g. cardiac MRI with stress testing), genetic testing, family history and endomyocardial biopsy are necessary and required by the TFC. Since TWI in the precordial leads are not very specific for ARVC/D, e.g. are commonly seen in patients suffering from coronary artery disease immediately after a coronary ischemic episode, and can persist for several weeks thereafter, it is important to exclude coronary ischemia by diagnostic tests such as coronary angiography or cardiac MRI.

### Limitations

Due to the observational and partially retrospective nature of this study, echocardiographic data was mainly obtained from previous reports. The duration of individual follow-up and lack of a follow-up ECG in some patients is a limitation that may have led to an underestimation of ECG changes. Variable ECG filtering may have affected the detection of epsilon waves, but not the detection of TWI, which was the main reversible ECG feature found in our cohort. Due to the retrospective study design our ECGs did not include right-sided chest leads (V1R, V2R, V3R, V4R), which could be included in future prospective studies investigating the diagnostic and prognostic utility of ECG in ARVC/D. Baseline and follow-up cardiac MRI were only available in a minority of patients, and thus associations between ECG and MRI findings could not be addressed in this study.

## Conclusions

This study supports the concept that ARVC/D is generally progressive, which can be detected by 12-lead surface ECG. Repolarization abnormalities may disappear during the course of the disease. Furthermore, the presence of TWI in inferior leads is common. Our findings support the use of 12-lead surface ECG to monitor disease progression in ARVC/D.
